# Impact of a Multi-Strategy Community Intervention to Reduce Maternal and Child Health Inequalities in India: A Qualitative Study in Haryana

**DOI:** 10.1371/journal.pone.0170175

**Published:** 2017-01-18

**Authors:** Madhu Gupta, Hans Bosma, Federica Angeli, Manmeet Kaur, Venkatesan Chakrapani, Monica Rana, Onno C. P. van Schayck

**Affiliations:** 1 Department of Community Medicine, School of Public Health, Post Graduate Institute of Medical Education and Research, Chandigarh, India; 2 Professor of Social Epidemiology, Department of Social Medicine, CAPHRI, Maastricht University, Maastricht, The Netherlands; 3 Department of Health Services Research, CAPHRI, Maastricht University, Maastricht, The Netherlands; 4 Department of Social Medicine, CAPHRI, Maastricht University, Maastricht, The Netherlands; TNO, NETHERLANDS

## Abstract

A multi-strategy community intervention, known as National Rural Health Mission (NRHM), was implemented in India from 2005 to 2012. By improving the availability of and access to better-quality healthcare, the aim was to reduce maternal and child health (MCH) inequalities. This study was planned to explore the perceptions and beliefs of stakeholders about extent of implementation and effectiveness of NRHM’s health sector plans in improving MCH status and reducing inequalities. A total of 33 in-depth interviews (n = 33) with program managers, community representatives, mothers and 8 focus group discussions (n = 42) with health service providers were conducted from September to December 2013, in Haryana, post NRHM. Using NVivo software (version 9), an inductive applied thematic analysis was done based upon grounded theory, program theory of change and a framework approach. Almost all the participants reported that there was an improvement in overall health infrastructure through an increased availability of accredited social health activists, free ambulance services, and free treatment facilities in rural areas. This had increased the demand and utilization of MCH services, especially for those related to institutional delivery, even by the poor families. Service providers felt that acute shortage of human resources was a major health system level barrier. District-specific individual, community, and socio-political level barriers were also observed. Overall program managers, service providers and community representatives believed that NRHM had a role in improving MCH outcomes and in reduction of geographical and socioeconomic inequalities, through improvement in accessibility, availability and affordability of the MCH services in the rural areas and for the poor. Any reduction in gender-based inequalities, however, was linked to the adoption of small family sizes and an increase in educational levels.

## Introduction

Large geographical and socioeconomic inequalities in maternal and child health (MCH) continue to persist in India and have even widened across states, between rural and urban areas and within communities [1]. Overall the maternal mortality ratio (MMR) in India is still as high as 1.67 maternal deaths per thousand live births [2] and the infant mortality rate (IMR) is 40 infant deaths per thousand live births [3]. IMR is higher in rural (44 per thousand live births) as compared to urban areas (27 per thousand live births) [3]. Socioeconomic MCH inequalities favoring the rich [4–6] and gender inequalities favoring male children [7] are also reported in India. To deal with MCH inequalities and improve MCH, the government of India implemented a multi-strategy community intervention known as the National Rural Health Mission (NRHM) from 2005–06 to 2012–13. NRHM’s aim was to improve the availability of and access to better-quality healthcare, especially for people residing in rural areas (to reduce geographical inequality), for the poor (to reduce socioeconomic inequality), and for women and children (to reduce gender inequality) [8]. Its health sector plans were health system strengthening; communitization (delegating powers to and empowering the community to monitor the health care delivery system) and specific maternal and child healthcare strategies [9]. [S1 File]. Details of these plans are given in a previously published study protocol [10]. NRHM aimed to reduce the IMR to 30 infant deaths /1000 live births, MMR to 1 maternal death /1000 live births.

This study is conducted in the state of Haryana in Northern India. It resembles other North Indian states in terms of socioeconomic development and sociocultural factors (like a strong preference for having sons, female feticide, a lower sex ratio, and a lower social status of women). At the same time, it provided a unique context by being a prosperous state with a rising economy, but with an unequal distribution of resources, leading to wide intra-state and inter-district differences in terms of the provision of basic infrastructure [11]. After the implementation of NRHM in Haryana, the MMR declined from 1.85 (2002–04) to 1.21 (2011–13) maternal deaths per thousand live births [2, 12] and the IMR from 59 (2005) to 40 (2012–13) infant deaths per thousand live births in Haryana [12, 13]. Quantitatively, MCH indicators improved considerably [14] and MCH inequalities between geographical regions (urban and rural areas), between rich and poor women (class differences), and between male and female children post NRHM were reduced [15].

However, these improvements were not uniform in the state, with certain districts performing better than others. It was not clear why the same strategy was working in one district, but not in the other. Neither was it clear what the pathways of change were for the overall improvement in MCH outcomes and reduction in inequalities. Knowing the pathway of change is crucial to understand the preconditions that need to be met before reaching the ultimate goal and to better understand the barriers and facilitating factors for the preconditions. This type of implementation research is paramount for the policy makers for an effective implementation of the NRHM health sector plans and meeting the intended goal of a reduction in maternal and child mortality and inequalities. NRHM is also continued in the second phase (2013–17) as part of National Health Mission [16]. There are limited studies estimating the effectiveness of interventions on equity in maternal or child health in low and middle-income settings [17]. Say and Raine (2007) highlighted the need to adequately grasp the contextual issues that must be addressed if inequalities in maternal health care use are to be reduced in developing countries [18]. Since such information cannot be obtained through quantitative surveys only, we planned a qualitative study.

Key areas of inequalities that are addressed in this study are geographical MCH inequalities between urban and rural areas, socioeconomic MCH inequalities between rich and poor and gender based child inequalities between boys and girls. It was envisaged that MCH plans of NRHM including health system strengthening, communitization, maternal health care strategies and child health care strategies would reduce these inequalities by improving the availability, accessibility and affordability of MCH services in the rural areas and for the poor women. This study aimed to explore the perceptions and beliefs of stakeholders (program managers, service providers, community representatives, mothers) regarding extent of implementation of these four major NRHM’s health sector plans; and effectiveness of these plans in improving MCH status and reducing geographical, socioeconomic and gender based MCH inequalities in two districts of Haryana in India with extreme situations, so as to have in-depth understanding of the contextual issues, barriers, facilitating factors, and the pathways of change for improving the MCH outcomes or reducing the MCH inequalities.

## Materials and Method

### Study area

Haryana has 21 districts with a total population of 25,353,081 (70% rural) and a birth rate of 21.3 per thousand mid-year population [3, 19]. The health care delivery system in Haryana has been described in the previous protocol study [10]. To obtain a better contextual understanding of two extreme situations and to learn which scheme works better in a particular situation, extreme case purposive sampling [20] was used to select one well-performing (district Ambala) [21] and one less well-performing district (district Mewat) [22] in terms of MCH indicators. (Table 1). Mewat is inhabited by Muslims (79%) predominantly, who are a religious minority group in India [23–25], while Ambala by Hindus (85%). Economy of Ambala is better than Mewat. Ambala has textile and surgical instrument industries in addition to agriculture as its source of income. Mewat is the least developed district in Haryana. Majority of its population resides in rural area and engaged in agriculture. There is scarcity of water in Mewat. The literacy rate of women is quite low (36.6%) as compared to males (70%). Women spends substantial amount of time in fetching the water and collection of wood for fuel. Only 20% of the villages have access to schools beyond primary level [24].

**Table 1:** Socio-demographic profile and maternal and child health indicators in district Ambala and Mewat.

District	Ambala	Mewat
**Population (N)**	1128350	1089263
**Rural Population (%)**	55	88
**Density**	717	723
**Average literacy rate (%)**	82	54
**Child 0–6 sex ratio**	810	906
**Standard of Living Index**		
**Low**	1.1	10.3
**High**	50.8	11.4
**Girls marrying before completion of 18 years (%)**	2.9	43.2
**Births of order 3 and above (%)**	21.7	65
**Teenage pregnancy (15–19 years) (%)**	0.9	9.3
**Received at-least 1 tetanus injection during last pregnancy (%)**	83	52.7
**Institutional births (%)**	55.4	14.8
**Post-natal care with in 48 hours of birth (%)**	70	33.7
**Fully immunized children (12–23 months) (%)**	79.1	11
**Children who received measles vaccine (12–23 months) (%)**	92	20.3
**Children with diarrhea who received oral rehydration solution (%)**	50	7.7
**Children had check up with in 24 hours after delivery (%)**	75.8	33.5

Source: Census 2011; Reference [21, 22].

From both Ambala and Mewat districts, we selected one village, one sub-center, one Primary Health Center (PHC), and one Community Health Center (CHC). As there was variability within the districts regarding MCH status, with certain blocks performing better than others, we purposively selected all well-performing and all poorly performing health facilities within the Ambala and Mewat district, respectively. Prior permission to conduct this study was obtained from the Mission Director, NRHM, Government of Haryana. The authors did not have any relation with those interviewed (like service providers) and the interviews were exclusively conducted for research purposes. The Ethics committee of the Post Graduate Institute of Medical Education and Research, Chandigarh, India approved the study.

### Study design

The theoretical framework underpinning this study was both grounded theory, i.e., to build theories from the data [26], and theory of change [27]. Theory of change is essentially a comprehensive description and illustration of why and how a particular change is expected to happen in a particular context leading to the desired goals. It defines long term goals and then maps backward to identify necessary preconditions so as to understand the pathway of change [28–29].

### Study population and data collection

The perceptions and beliefs of the participants were explored using pretested focus group discussion and in-depth interview guides. (S2 File). Focus group discussions were conducted separately with different MCH service providers: accredited social health activists, auxiliary nurse midwives, medical officers, or senior medical officers in each district. Each focus group discussion had about 4–10 participants. In-depth interviews were conducted with the Mission Director NRHM, MCH program managers at the state and district level, the community leaders and mothers at each level (ie., village, subcenter, PHC, CHC and district). Qualitative interactions were continued until data saturation. After obtaining written informed consent, all the interviews and discussions were audio and video-recorded and field notes prepared. Repeat interviews were conducted with program managers at the state level. The duration of the focus group discussions ranged from 60 minutes to 90 minutes and in-depth interviews ranged from 45 minutes to 60 minutes. Two female authors (a doctor with an MD in Community Medicine and a research scholar with a Master degree in Public Health), who were trained in qualitative research methods, collected the data from September to December 2013 (post-NRHM period). Focus group discussions and in depth interviews with the participants were conducted in their place of choice. Only authors, study staff, and respective participants were present at the time of data collection, so that participants were comfortable; privacy and confidentiality were ensured. No one refused to participate in the study. However, one community representative could not be interviewed because of his unavailability at the time of data collection.

### Data analysis

**Fig 1:**
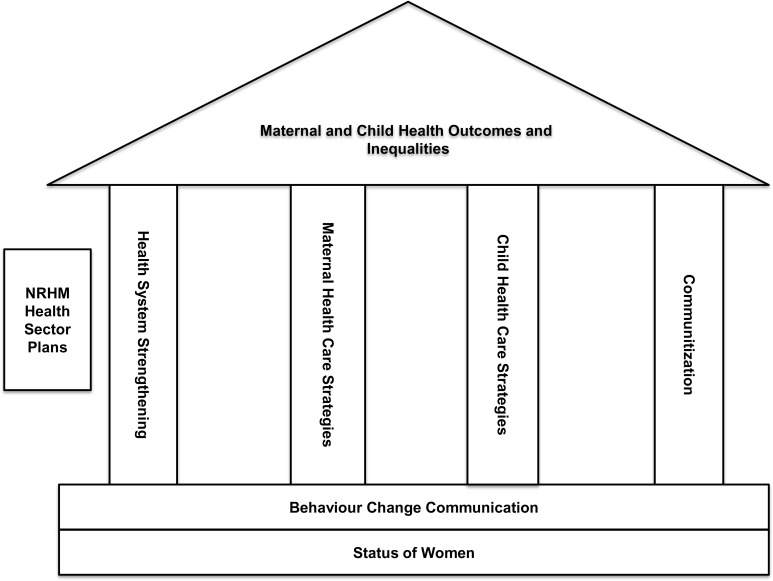
A conceptual framework of NRHM.

Audio and video recorded focus group discussions and in depth interviews were transcribed in Hindi and translated into English. Two independent coders (authors) coded the translated text for grouping into categories. A conceptual framework of NRHM was used during the analysis. As per this framework, NRHM’s health sector plans had four major pillars–health system strengthening, communitization, maternal health care strategies, and child health care strategies–to improve MCH and reduce MCH inequalities. (Fig 1). Fundamentals were the behavior change communication of the community and the social status of women, as these were the determinants of MCH outcomes.

Using NVivo software (version 9), an inductive applied thematic analysis [30] was done based upon grounded theory [26], program theory of change [27] and framework approach [31]. In addition to the pre-determined codes (as listed in the focus group discussion/in-depth interview guide), codes emerged from the analysis of focus group discussion and in-depth interviews (‘emergent codes’). Since, one of the key objectives was to compare the MCH status and inequalities in district Ambala and Mewat, hence, based on these codes themes were identified and compared between the two districts. The framework approach was used to align the identified themes and codes as per the conceptual framework of NRHM. (S3 File). The program theory of change was applied to construct pathways of change by identifying the necessary preconditions that led to early, intermediate and long-term changes in the community (benefitting the NRHM goals).

## Results

A total of 33 in-depth interviews (n = 33) with program managers, community representatives and mothers, and 8 focus group discussions with service providers (n = 42) were conducted. The background characteristics of the participants are given in Table 2. The participants’ mean age was 34.8 years. Forty percent of the participants were between 20 and 29 years and 63% were females. Mean years of experience in the health system varied from 3 years for accredited social health activists to 10 years for auxiliary nurse midwives.

**Table 2:** Background characteristics of the participants of focus group discussions and in-depth interviews.

Characteristics	Focus Group Discussion	In-depth Interviews	Total
	N = 42 (%)	N = 33(%)	N = 75(%)
**Mean age of participants**	35.4 years	34.1 years	34.8 years
**Age in years**			
20–29	14 (33.3)	17 (48.4)	31 (39.7)
31–39	14 (33.3)	4 (9.7)	18 (23.3)
40–49	9 (21.4)	11 (35.5)	20 (27.4)
50–59	5 (11.9)	1 (3.2)	6 (8.5)
**Sex**			
Female	24 (57)	24 (71)	48 (63)
Male	18 (43)	9 (29)	27 (37)
**Education**			
Illiterate	0	6 (12.9)	6 (5.5)
Primary	3 (7)	4 (12.9)	7 (9.6)
Middle	2 (4.8)	0	2 (2.7)
Matric	7 (16.7)	6 (19.4)	13 (17.8)
Senior Secondary	5 (11.9)	1 (3.2)	6 (8.2)
Graduation and Post Graduation	4 (9.5)	9 (29)	13 (17.8)
Professional	21 (50)	7 (22.6)	28 (38.4)
**Occupation**			
Laborer	-	1 (3.2)	1 (1.4)
Housewife	-	17 (48.4)	17 (20.5)
Community leader	-	6 (19.4)	6 (8.2)
Auxiliary nurse midwife	10	-	10 (13.7)
Accredited social health activist	11	-	11 (15)
Doctor	21	-	21 (28.8)
Program Manager		9 (29)	9 (12.3)
**Mean years of experience in the health system**			
Auxiliary nurse midwife			9.9
Accredited social health activist			2.7
Doctors			7.6
Program Managers			7.5

Overarching themes, subthemes and codes are given in S1 File. Implementation status of MCH plans of NRHM and their effectiveness in improving MCH outcomes and reducing geographical, socioeconomic and gender based MCH inequalities as per the interviews and focus groups discussion with stakeholders in district Ambala and Mewat is described below, and summarized in S3 File.

### Implementation status of MCH plans in district Ambala and Mewat, Haryana

#### Health systems strengthening

**Infrastructure strengthening.** Almost all the participants reported that during the last 2–3 years of the NRHM period, the health infrastructure improved in terms of the availability of cleaner and well-equipped health centers providing MCH services. This improvement was perceived to be more in Ambala as compared to Mewat district. However, few of the mothers in the villages in district Mewat had reported that nothing had changed over the years in that district.

“Infrastructure has improved a lot, earlier it was negligible. Only during NRHM, new born corners and stabilization units were established” (Program manager, Ambala)

However, there were still insufficient numbers of health facilities as per the population norms. It was reported that many centers lacked certain diagnostic facilities (in CHCs) and waiting halls for patients (in sub centers and PHCs). According to some auxiliary nurses, the lack of toilets in some facilities posed a barrier to collect urine samples of pregnant women.

“There should be one CHC on one lakh population. We are running CHC at one lakh 45 thousand. There should be more PHCs to decrease the workload of CHC”. (Medical officer, Ambala).

“There is no facility of ultrasound and x-ray in the village. We are paying outside for these facilities. Only facility of normal delivery is here. I had a delivery in the 8th month through operation. At that time, they referred me to a private hospital from here [government] as there was no machine to keep the baby.” (Mother, Mewat)

This indicated that there was partial and non-uniform improvement in the health infrastructure in the districts. Existing health centers were catering to larger population beyond their expected population catchment norms. Infrastructure development was reported more in Ambala district as compared to Mewat district. Hence, some improvement in MCH outcomes and bridging of geographical MCH inequality between urban and rural areas could be expected due to infrastructure development.

**Drugs and logistics.** Service providers, program managers, and mothers reported that free medicines were available in the public health facilities during the NRHM implementation in both Ambala and Mewat district. As a result, the number of patients in the health facilities had increased. Not only this, but it had also improved the health of poor mothers and children.

“Free medicines have helped the poor mother and children in getting the treatment from the government hospitals; hence, they now remain healthy….” (Community leader, Ambala).

However, out of stock situations and less faith in medicines prescribed for children in the government sector prevented its access especially in district Mewat. Most of the mothers, accredited social health activists and nurses in district Mewat have reported that families got the treatment of their children from the private hospitals, in-spite of availability of free medicines and free treatment in the public hospitals because of their perceived belief that these medicines were not of good quality.

Availability of free medicines under NRHM had improved the affordability of the MCH services especially for the poor families. This definitely had implications on bridging the socioeconomic MCH inequalities. However, perceived lack of faith on the quality of medicines in the public health facilities was an important barrier to access these freely available medicines, especially in district Mewat.

**Patients transport service (Free ambulance service).** The service providers and program managers perceived that the free ambulance service was a major factor contributing to the increase in institutional deliveries in district Ambala and Mewat. The ambulance dial number was widely disseminated among the villagers in the district Ambala. According to the interviewees, through the accredited social health activists in the villages, the utilization of the ambulance by the mothers had also increased.

“The increase in institutional deliveries is all because of the ambulances. Earlier there were 5–7 deliveries at our CHC (in 2005) and then it was increased to 15 deliveries in 2011. Now today (2013) there are 45 plus deliveries (per month).” [Medical officer, Ambala].

“We dialed 102 and ambulance came to take us to hospital”. [Mother, Ambala]

However, in the district Mewat, there was less awareness about the ambulance’s free availability and the dial number. Also, there was either a delay in reaching the remotest areas, or it did not reach it at all, especially at nighttime, or the dial number could not be reached, possibly due to frequent callers. This resulted in less faith and more home deliveries. Shortage of ambulances was reported from district Mewat; and the problem of maintenance of these vehicles was reported from both the districts.

This indicated that patient referral services had increased the access to basic emergency obstetric care services in the rural areas, and hence might have contributed in reducing the geographical inequality in institutional delivery rates in urban and rural areas in district Ambala. However, this improvement was not so much reported from district Mewat due to problems in accessing the referral service.

**Human resources.** It was perceived by the senior medical officers, that the availability of doctors, auxiliary nurse midwives, staff nurses increased during the NRHM period, but simultaneously that the demand of services had increased manifold especially in district Ambala. This had led to acute shortages of manpower. The shortages of doctors had also overburdened the existing staff and resulted in a poor access to health care services.

“There were earlier 10–15 patients in the outpatient department, now there are up to 150 patients in outpatient departments. In 2005, we (PHC) didn’t have deliveries and in medical college (private medical college in the district), there used to be 150 deliveries, but now (year 2013) we have 600 and they have only 200 deliveries”(Medical officer, Ambala).

“Here, work of four people is done by one person. Then how can that person do so much work, and obviously his efficiency will suffer.”(Auxiliary nurse, Ambala).

The lack of specialists had forced the non-specialist doctors to treat seriously ill patients. This compromised the quality of health care or led to referrals to other health facilities with additional health risks during transportation. It was also expressed that existing specialists were not meaningfully distributed with some districts having many specialists and others lacking them (e.g., district Mewat). The situation is even worse in the Mewat district, as ground level workers, such as the auxiliary nurse midwives, were not allegedly available in all the sub centers: hence, the provision of MCH services in such villages was not optimal.

“There are 86 sanctioned posts for medical officers in Mewat, but there are hardly 30–32 doctors.” (Medical officer, Mewat).

“There is no surgeon here; no skin specialist is here, no radiologist here. …Nobody is here.” (Medical officer, Mewat).

“We have one auxiliary nurse on a population of 12,000; now there is one auxiliary nurse on a population of 16,000 and in some villages there are no such nurses at all.” (Auxiliary nurse, Mewat).

A program manager at the Mewat district expressed his concerns about the frequent transfers of trained staff to their native districts in spite of getting extra financial incentives. This not only led to the loss of human resources, but also the loss of money and too much time invested in their trainings.

“The attrition rate is very high in Mewat. Doctors are not willing to join at the salary we are giving.” (State-level NRHM officer).

There was a provision for hiring contractual staff to manage the acute shortage of staff. However, medical officers expressed that the contractual staff lacked proper training and skills to provide quality MCH services. They were also less paid as compared to regular staff, which demotivated them. Furthermore, as reported by village health activists, some pregnant women were afraid to go to PHCs for delivery due to the perceived lack of an adequate number of nurses, the negative and casual attitude of doctors, and the physical abuse of pregnant women in the labor room.

“She delivered a baby girl, but after one hour she started bleeding; doctors then were having lunch and then they did not take care and she died.” (Auxiliary nurse, Mewat)

Hence, it was observed that availability of sufficient number of skilled and trained service providers in the public health sector was still the greatest challenge in both the districts. However, the shortage of human resources was much more in district Mewat as compared to district Ambala. NRHM’s plans had the provision of hiring additional contractual staff, but the requirement was much more. However, the contractual staff was not well trained, and their attrition rate was very high especially in district Mewat. There was also unequal distribution of health specialists in the districts.

**Untied funds.** Untied funds were perceived to be useful for upgrading the infrastructure and other need-based support by medical officers in district Amabala. In Mewat, these funds were not reported to be sufficient by medical officers. Participants also expressed that the stringent accounting formalities and the lack of awareness regarding the accounting procedures were substantial barriers for an effective utilization of these funds in both Ambala and Mewat district.

**Medical mobile unit.** The medical mobile unit, intended to cater the MCH needs of the hard-to-reach areas in the Mewat district, was believed to be non-functional, possibly due to the lack of doctors.

“Medical Mobile Unit has zero role… There are no doctors in mobile units. Or they [authorities] could not appoint doctor. No service nothing… No man power is there.” (Medical officer, Mewat)

Mobile medical units could not bridge the geographical MCH inequalities between difficult to reach areas and easily accessible areas in district Mewat, as these were non-functional and inaccessible due to lack of doctors.

#### Communitization

**Accredited social health activists.** Of all the schemes under NRHM, the accredited social health activist’s scheme was the most appreciated scheme by all the participants in both the districts. Notably, some auxiliary nurses who were closely working with health activists at the sub-centers believed that these activists were educating the beneficiaries about MCH schemes and supporting them in conducting immunization sessions, mobilizing the children and pregnant women on the immunization session day, providing antenatal care, and motivating pregnant women for institutional deliveries. Similarly, many mothers stated that, primarily through the activists, they had understood about the importance of immunization, institutional delivery, and the possible adverse negative effects of home delivery. These activists had also supported them during delivery and assisted them in availing the free ambulance service, the treatment in the health facilities and the financial incentives for getting the institutional delivery. In particular, activists perceived to have played a vital role in convincing potential troublemakers in the families [often mother-in-laws] to ensure hospital care for pregnant mothers. They seemed to have established an excellent rapport with them.

“She has made me understand that delivering a baby at home is not good as the child might die during labor” (Mother, Mewat). “As far as the percentage of institutional delivery is concerned, presently it is 80% to 90%. In promotion of these institutional deliveries, social health activists have played a major role.” (State NRHM officer).

In Mewat, the program manager shared that large number of positions were vacant due to the lack of potential women candidates meeting the educational requirements even after the relaxation of the minimum education level from the 8^th^ to 5^th^ grade. However, medical officers reported that some activists faced challenges in understanding their job description possibly due to low educational status.

“For Mewat, we have have asked the district authority to relax the minimum qualification of activists from matric/eight to fifth class. But even after this, we were not able to recruit fifth passed activists because the illiteracy rate is very high in Mewat.” (Program Officer, Mewat)

Accredited Social Health Activists scheme was reported to be the most effective schemes in terms of reducing MCH geographical and socioeconomic inequalities between urban and rural areas and between rich and poor. She was perceived to be involved in mobilizing the community through behavior change communication with decision makers in the families to utilize the MCH services. She was also perceived to have increased the accessibility of MCH services by establishing effective linkages between the community and public health system in both the districts. However, because of low education status of women in Mewat, there were problems in appointing sufficient number of activists in the villages. This might be the one of the reasons for low utilization MCH services in this district and poor MCH outcomes.

**Village health nutrition day.** Although most mothers and community leaders did not know about this day, some were aware of the village health melas (fair), which were conducted along with the immunization camps in both the districts. As reported by some medical officers and state-level NRHM officers, the village health nutrition days were not conducted regularly mainly due to the inadequate number of auxiliary nurses.

“This day is combined with the immunization session. Auxiliary nurse midwives have lot of work on Wednesday, like vaccination etc. So I don’t think she tells anything. She only ticks marks on papers. (Senior medical officer, Ambala)”

This scheme was perceived to be not implemented well, and also that it did not contribute in improving the MCH outcomes in villages or bridging the geographical inequalities.

**Village health nutrition and sanitation committee.** The medical officers shared that, although such committees were constituted in many villages, these were not involved in the planning and implementation of activities in both the districts. A medical officer reported that some village heads [presidents] were expecting ‘some money’ [bribe] from the allocated committee funds (154 USD) for the infrastructure and program-related activities. As a result, auxiliary nurses were hesitant to withdraw the funds from the joint bank account and these remained unutilized. The involvement of grass-root worker from women and child development department (member) in the operationalization of the bank account was also a barrier in the utilization of funds, due to a lack of inter-sectoral coordination. However, few ASHA’s in district Mewat had reported that these committees utilized the funds to clean the villages.

“*The funds of the committee are not utilized properly from the time the account has been shifted to Anganwadi workers*. *Earlier it was with village health activists*, *but now it is with Anganwadi workers and they don’t spend the budget of Rs 10*,*000 properly*. *So the account either should be with activists or with the auxiliary nurse*. *Implementation of this scheme is only around 30–40%*.*”* (Senior medical officers, Ambala)

This scheme did not perceived to be implemented well in the villages in both the districts.

#### Specific maternal and child health schemes

**Financial incentive scheme for institutional delivery (*Janani Suraksha Yojana*).** Many mothers and community leaders had an inadequate knowledge about this scheme and believed that some cash benefits were given to the poor mother for delivery. Almost all the participants had reported delayed payments to the mothers due to stringent administrative procedures, such as the need for the submission of several supportive documents (e.g., poverty line card, aadhar card—an unique identification number card) in both Ambala and Mewat district. Most pregnant women did not have some of these documents (e.g., bank account in the name of the pregnant woman) especially in district Mewat.

This scheme was reported to have contributed in increasing the institutional delivery rate especially in rural areas in district Ambala and Mewat, and hence had contributed in bridging both geographical and socioeconomic inequality to some extent. However, with the change in the guidelines of its implementation, such as its linkage with opening of bank accounts, this scheme had suffered a set back.

**Scheme for free delivery and free treatment of pregnant women and infant in the public hospitals (*Janani Shishu Suraksha Karyakaram*).** Although most of the mothers and community leaders were not aware of this scheme’s name, they were aware of the free delivery possibility and the free treatment of mothers and infants in public hospitals in both the districts. Service providers believed that due to the facilities provided under this scheme, mothers preferred to go to the government hospitals for delivery and therefore the institutional deliveries had increased. Keeping them in the hospital for at least 48 hours after a normal delivery was, however, an issue, as mothers usually preferred to go back to their homes within 24 hours to meet their household responsibilities. In this relation, doctors were not able to motivate them, partly due to an inadequate number of post-partum beds. The medical officers believed that MCH services were now affordable for poor people and the shortage of human resources prevented the full implementation of this scheme in both the districts.

This scheme was reported to have lot of potential in improving MCH and bridging socioeconomic and geographical MCH inequalities due to its provisions of free delivery and free treatment of sick children in both the districts. However, lack of sufficient number of doctors in the health facilities had prevented its full impact, especially in district Mewat.

**Immunization.** Many mothers reported that they usually get immunized during pregnancy and have their children immunized too. However, inadequate numbers of auxiliary nurse midwives for immunization, cultural barriers against immunization (not considered safe by some members in the community), and fear for injections, especially in the Mewat district, were expressed as barriers for immunization. It was reported by mothers in Mewat that they would rather get their girl child immunized than the male child, because of the perceived belief that immunization was not safe for children. The medical officers reported that the provision of alternate vaccine delivery (where there were no auxiliary nurse midwives) and the involvement of religious leaders to help deal with the cultural barriers in Mewat resulted in an improved immunization status.

Gender based differences in immunization were not reported in district Ambala. These were reported from Mewat, which rather had favored the girl children as more number of girls was receiving vaccines as compared to boys.

**Facility based newborn care.** Facility-based newborn care services in public hospitals were believed to have improved drastically during NRHM and they were considered better than those in private hospitals in district Ambala. In district Mewat, it was reported that facility based newborn care services had improved, but non-availability of 24X7 staff prevented its proper implementation.

“All the districts now have sick new born care units, the infrastructure of which has been better than is available in the private sector. As a result many of sick newborns are being referred to government hospitals for treatment.” (State Child Health Officer)

**Integrated management of neonatal and childhood illnesses.** State program managers reported that, although medical officers and auxiliary nurse midwives were trained in the integrated management of neonatal and childhood illnesses, it was not implemented properly due to inadequate human resources and supervision, and shifting of focus on implementing home based post natal care scheme by accredited social health activists in both the districts.

### Barriers in accessing MCH services in district Ambala and Mewat

As per service users and medical officers, the client-level barriers were: poor awareness of mothers about NRHM’s schemes, overriding household responsibilities of mothers that prevented post-delivery care in the hospitals, phobia towards injections or operations that prevented access to seek immunization, and emergency obstetric care (especially in the Mewat), lack of faith in medicines supplied in government hospitals (especially for the treatment of sick children), and unmet basic needs like not having enough food for pregnant women and children from poor families. In Mewat it was reported that health facilities were not located at the strategic locations and hence were less accessible. Community-level barriers were: low social status of women in the society (especially in the Mewat), lack of decision making power of the mothers to seek MCH care (as their mothers-in-law and spouses were the main decision makers), gender disparities in providing childcare with some families preferring male children’s health care above female’s health care, cultural barriers among some religious minority communities that discouraged institutional deliveries (possibly because of the lack of adequate female doctors), immunization, and the uptake of family-planning methods. The lack of willingness of the community members to wait for their turn to see a doctor in the government health facilities was another community-level barrier, although it is linked to inadequate human resources and high patient load in public hospitals. Program manager expressed that there was lack of political will to do the overall development in Mewat district, because they would not get additional grant of funds if its current status changed.

The differences in the implementation of MCH sector plans of NRHM in district Ambala and Mewat are summarized in Table 3. Plus signs denote the perceived level of status of implementation of NRHM health sector plans, or of prevailing barriers in the implementation of these plans in district Ambala and Mewat. Hence, single plus ‘+’ sign indicates perceived fair implementation, double ‘++’ good implementation and triple ‘+++’ very good implementation. For the barriers in the implementation of these plans, single plus ‘+’ sign indicates perceived small barrier, double ‘++’ moderate barrier and triple ‘+++’ large barrier. Negative sign ‘-’ indicates either perceived no implementation of the MCH plan or no barrier, respectively.

**Table 3:** Differences in implementation of MCH sector plans of NRHM in district Ambala and Mewat.

NRHM health sector plans	Ambala	Mewat
**Health system strengthening**		
Infrastructure	+++	+
Manpower	+++	+
Drugs and logistics	+++	++
Mobile medical units	not applicable	+
Referral transport	+++	+
**Communitization**		
Accredited Social Health Activists	+++	+
Village health nutrition and sanitation committees	+	+
Village health nutrition days	+	+
**Maternal Health care strategies**		
*Janani Suraksha Yojna*	+	+
*Janani Sishu Suraksha Karayakaram*	++	+
24X7 delivery points	++	+
**Child health care strategies**		
Facility based newborn care	++	+
Integrated management of neonatal and childhood illnesses	+	-
Immunization	+++	+
**Barriers**		
Cultural Barriers	-	+++
Illiteracy	-	++
Awareness about schemes by mothers	+	+++
Hard to reach areas	-	+
Areas with no connectivity by road	-	+
Lack of specialists	+	+++

### Effectiveness of MCH plans in improving MCH outcomes and reducing MCH inequalities

It was perceived by most of the participants that maternal deaths have reduced in the last 4 to 5 years mainly due to increased institutional delivery, availability of accredited social health activists and also due to better management of anemia and tetanus immunization during antenatal period in district Ambala and Mewat. However, it was reported from district Mewat that there were some instances of home deliveries by unqualified persons that had led to maternal deaths. Infant and under five mortality and morbidity was reported to have decreased in both Ambala and Mewat districts. However it was reported from Mewat district that because of no adoption of family planning methods and unavailability of pediatrician in the public health facilities the child health had suffered. Availability of MCH services was reported to have improved in both the districts but there was acute shortage of manpower. Affordability of MCH services was reported to have increased in both the districts due to free medicines, free treatment of sick children and provision of cashless delivery and free referral transport services.

Regarding geographical MCH inequalities, most of the participants expressed that there was increase in the availability and access to health facilities, doctors, and medicines in rural areas during the NRHM period. Hence, they believed that MCH inequalities between urban and rural areas had reduced to some extent. However, regional differences remained between remote villages and urban areas. Most of the study participants believed that the NRHM, through provision of free government services, had played a significant role in addressing the socioeconomic inequalities between the rich and poor. Regarding the gender inequality, it was believed that the government alone could not deal with gender inequalities; it had to be dealt with at the societal level by changing the attitude of society towards female children, the adoption of small family sizes, and by increasing educational levels.

Overall, the perception of program managers, medical officers, auxiliary nurse midwives and accredited social health activists did not differ much regarding the implementation and effectiveness of the NRHM’s health sector plans. However, although they often did not know the exact name of the schemes, community representatives and mothers were more aware of the visible benefits of the NRHM’s schemes like health activists in the villages, free ambulance services, free institutional deliveries, and free treatment of sick newborn in the public hospitals.

The key points of this study are summarized in Table 4.

**Table 4:** Summary table of key points of status of implementation of MCH plans of NRHM and its effectiveness in reducing geographical, socioeconomic and gender based MCH inequalities.

Implementation Status of MCH plans of NRHM	Key Findings
**A. Health system strengthening**	
Infrastructure	Availability of the well equipped health facilities in rural areas in the last 4 to 5 years have improved the access to MCH services in rural areas that might have bridged the geographical inequalities in urban and rural areas
Drugs and Logistics	Free availability of medicines in health centers in rural areas has improved the affordability of MCH services, which might have reduced the socioeconomic inequality between rich and poor. However, perceived lack of faith in the quality of these medicines reported by the mothers that prevented their access especially in district Mewat.
Patient Transport Service	Free availability of ambulance service was linked to increase in access to MCH services especially for institutional delivery, which might have contributed in reducing the geographical MCH inequality in urban and rural areas. However there were issues with its maintenance, and better services with in the ambulance were needed at par with private. Other problems like inadequate number of vehicles, ambulance contact numbers could not be reached possibly due to frequent callers or late arrivals to the homes especially in district Mewat were reported to have resulted in home deliveries.
Human resource	Acute shortage of manpower was reported especially specialist in both the districts. This problem was reported more in district Mewat, because of higher attrition rate of staff and non-uniform distribution of specialists with in the state. NRHM contractual staff was available but quality of contractual staff was an issue. Shortage of human resources prevented good quality of, availability of and access to MCH services especially in district Mewat.
Untied funds	These funds were reported to be very helpful for upgrading the infrastructure in the health facilities or buying drugs as per the need or utilizing these funds for arranging refreshments for mothers during mother’s meetings. These funds reported to have empowered the service providers to meet their needs at the local level.
Mobile Medical Units	Since functional status of mobile medical units was reported to be an issue mainly due to non-availability of doctors and limited awareness of mobile medical units in the villages in district Mewat, its basic purpose of increasing the access in the hard to reach areas did not seem to have met.
**B. Communitization**	
Accredited Social Health Activists	All the stakeholders appreciated this scheme. It was believed that she was a community mobilizer and played an important role in immunization of children and pregnant women, improving institutional delivery, generating awareness about NRHM schemes & importance of institutional delivery in the villages. Because she was well known in the villages, had good rapport with the women especially decision makers (mother in laws), she called free ambulance and accompanied the families to the hospital for institutional delivery. She acted as a bridge between community and health facilities and improved the access to MCH services in the rural areas and contributed in reducing the geographical MCH inequalities. However, minimum educational qualification has a bearing on recruitment of accredited social health activists especially in district Mewat.
Village Health and Nutrition Day	These were known popularly as village health ‘*mela’*. Immunization sessions and mother meetings were held on village health and nutrition days. These were not held regularly and did not contribute much in increasing the availability or accessibility of MCH services.
Village Health Nutrition & Sanitation Committee	Members of this committee were not involved in need based village health planning. Village head would ask for bribe for utilizing the funds; and involvement of *anganwadi* worker in funds handling led to underutilization of funds.
**C. Maternal Health Care Strategy**	
*Janani Suraksha Yojna* (Financial incentive for institutional delivery)	This financial incentive scheme was reported to have increased the institutional delivery rate and improved the affordability for utilizing MCH services. However, there was delay in payment to the pregnant women due to administrative reasons. Linking the disbursements of financial incentives with opening of bank accounts in the name of pregnant women had resulted in underutilization of funds under this scheme, due to lack of proofs with the pregnant women that were required to get the bank account opened.
*Janani Shishu Suraksha Yojna* (Free medicine, treatment and institutional delivery)	Availability free diet during hospital stay and cash less delivery in the health facilities was linked with increased institutional delivery. This scheme was also reported to have increased the affordability of MCH services and might have contributed in reducing the socioeconomic inequality between rich and poor. However, implementation of this scheme was reported to be partial due to lack of adequate manpower.
**D. Child health care strategies**	
Immunization	Lack of sufficient auxiliary nurse midwives had led to the partial implementation of immunization sessions. Cultural barriers like fear of injections were reported for immunization of children especially in district Mewat. Accredited social health activists were reported to be the catalyst in improving the immunization coverage by mobilizing the mothers and family members.
Facility based newborn care	Newborns were reported to be referred for treatment to government hospitals from private health facilities, as government new born facilities were better. This might have contributed in reduction in infant mortality rate.
Integrated management of neonatal and childhood illnesses	Staff was trained in integrated management of neonatal and childhood illnesses implementation. However, caregivers lacked trust on government facilities for treatment of sick children so they did not visit subcenters in villages for treatment (less demand at subcenter level). Also due to lack of supervision of trained staff there was poor implementation of this scheme. Hence the focus of implementation was shifted from integrated management of neonatal and childhood illnesses scheme to home based postnatal care scheme.
**Effectiveness of MCH plans in improving inequalities**	
Geographical Inequality between urban and rural areas	It was perceived that due to increase in utilization of MCH services in the villages in rural areas in the form of increase in antenatal registrations, institutional deliveries, reduction in maternal and infant deaths the gap between rural and urban areas regarding MCH services was bridged to some extent due to implementation of NRHM health plans. However it was reported that facilities were still more in cities.
Socioeconomic Inequality between rich and poor	Socioeconomic inequalities were perceived to have decreased to some extent because of availability of free ambulances, medicines, and diet during hospital stay for the poor. However, it was reported that food security in general would reduce this.
Gender Inequality between girls and boys	It was believed that NRHM had no scheme for targeting gender inequality. Small size of the families and increased educational status reported to have led to the changes in gender inequality; Gender inequality was less seen in Mewat district

## Discussion

Starting from the four major pillars of the NRHM (health system strengthening, communitization, and specific maternal and child health strategies) to improve MCH and reduce MCH inequalities, service users and other stakeholders much appreciated the role of health systems strengthening, especially for the provision of free ambulance services and free medicines in public hospitals. Communitization efforts were also much appreciated, particularly for the availability of accredited social health activists at the village level. It was reported that, under specific MCH strategies, free MCH services, such as antenatal care, institutional deliveries, and the treatment of sick children, were also implemented better. This held particularly for the well-performing Ambala district. Stakeholders believed that overall MCH outcomes had improved during the NRHM implementation and that maternal and infant mortality had also declined, as had geographical and socioeconomic MCH inequalities. These perceptions correspond well with the quantitative data of the district-level household survey Haryana for the year 2012–13 [14] and a recent study on effectiveness of NRHM in reducing MCH inequalities [15].

**Fig 2:**
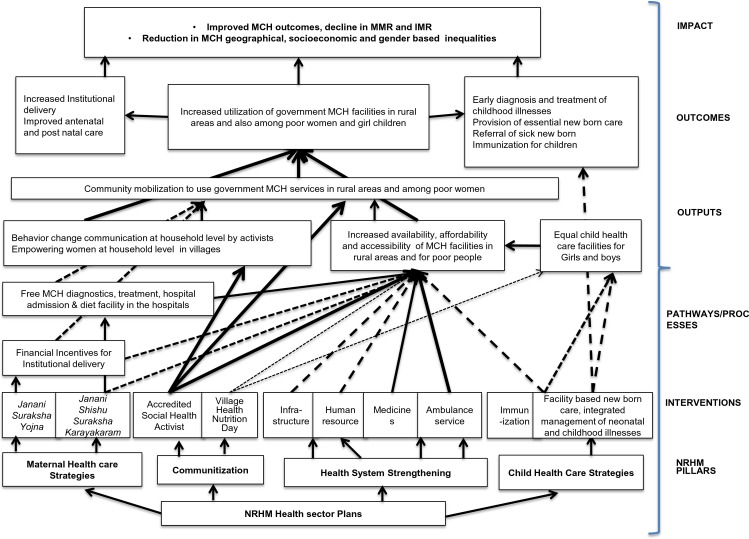
The pathways for change as derived from the theory of change.

The pathways for change, as derived from the theory of change, which might have led to the improvements are depicted in Fig 2. The NRHM’s four pillars and corresponding interventions are depicted at the bottom. The arrows from the interventions lead to the boxes that represent the outputs. The weight of the arrows (dotted vs full) indicates the intensity of the effect of that intervention on the outputs. Weight is decided based upon the perceptions of the participants regarding an intervention. For example, the weight for accredited health activists is denoted by full arrow, as this intervention is perceived to be very effective in improving the MCH outcomes as compared to village health nutrition day (denoted as dotted arrow). The arrows from the outputs lead to the outcomes and finally to the impact at the top. As per the theory of change, NRHM’s interventions are the preconditions (inputs) to produce the outputs, the outcomes, and the impact, respectively. The outputs are relatively immediate effects that are expected to happen after inputs and processes that add more details in relation to the product of the activity e.g., number of persons trained in sick child management. Outcome indicators refer more specifically to the objectives of an intervention, that is its ‘results’ e.g., management of sick child; and impact indicators refer to the health status of the target population that do not show progress over relatively short periods of time like child mortality rate [32].

An important precondition to achieve the NRHM’s goal of reduction in IMR and MMR and MCH inequalities was the delivery by skilled birth attendants, especially in rural areas and for poor women. Delivery by skill birth attendants was ensured by the institutional deliveries. The pathways for change, which might have led to the increase in institutional deliveries, included the availability of accredited social health activists in the villages, who did behavior change communication with mothers and potential influencers in the family. This empowered the pregnant women with enough knowledge regarding the health sector plans of NRHM (free ambulance services, free hospital deliveries, free neonatal treatment, and financial incentives for hospital deliveries) and enabled them to take decisions regarding institutional delivery. She acted as a bridge between the community and public hospitals. As a result, the community was mobilized to use the MCH facilities in rural areas. These factors, along with the availability of health facilities and doctors in rural areas and the free ambulance service, free medicines further improved the accessibility and affordability of MCH services and benefitted poor pregnant women and children. The pathways of change constructed in this study can be supported by various other studies. Nonyane et al (2015), reported that community-based interventions delivered by female community health volunteers can be instrumental in improving equity in levels of facility delivery and other newborn care behaviours [33]. Brazier et al (2015) reported that building the capacity of community-level cadres to promote maternity care-seeking by women in their villages is an important complement to facility-level interventions when increasing the availability, quality and utilization of essential maternal health services [34]. Bridging inequalities through an analytical framework, Jacob et al (2011) reported that the combination of interventions in NRHM is required to tackle barriers in health care access [35]. Parkhurst and Ssengooba (2009) reported that if the mothers are properly counseled and mobilized and given the enabling environment, in terms of the ambulance service and the accompanied person for the institutional delivery, they will go to the local functional health facility and not bypass it [36]. In a systematic review by Yuan et al (2014), it was concluded that interventions that were effective in reducing inequity included the improvement of health care delivery by outreach methods, using human resources in local areas, using services in the community nearest to the residents, and providing financial incentives or knowledge support to the demand side [17].

The preconditions in the pathway of change, however, were not fully met in the Mewat district. This was due to health system barriers, like the inadequate number of accredited social health activists for community mobilization, the lack of auxiliary nurse midwives and doctors in the hospitals to provide MCH services, and a poor ambulance service to improve the access. This was compounded by the presence of client and cultural barriers for accessing MCH services. An acute shortage of manpower was perceived as the major health system barrier in accessing quality MCH services. Shaw et al (2015) also reported that lack of health extension workers is the most common barrier to the utilization of sick childcare services [37]. The underlying reason for the poor MCH status in the Mewat district was also the perceived lack of political will to improve the basic socioeconomic conditions (structural determinants of health inequalities), despite the special status granted to the district and extra financial contributions by the government of India [38]. Interventions, that are otherwise well designed, therefore, might not have work. An enabling environment at the structural, political and cultural level is an important precondition. Thus, as depicted in Diderichsen’s model, the health system acts as an intermediary determinant of health inequalities [39]. Structural determinants in the social and political context (giving rise to unequal socioeconomic positions, income, education, power) are the major social determinants of health inequalities [40]. These need to be tackled to reduce health inequalities. Pallikadavath et al (2013) reported that districts that provide good connectivity by roads, better educational facilities for children, and recreational facilities have better retention of human resources for MCH care (compared with districts providing financial incentives) [41]. Fleuren et al (2004) in their review on determinants of innovation in health care organization reported that characteristic of organizations, the users adopting the innovation, the innovation, and the socio-political context are the important determinants of a successful implementation of an innovation [42]. Hence, it can be learnt from the Mewat experience that, for an effective implementation of MCH plans, the overall socio-economic development in sectors such as education, employment, infrastructure development, and social welfare also need to be addressed.

Based on the participants’ perspectives, NRHM did not seem to have contributed much to the reduction in gender inequalities, except for the measures related to immunization. The better immunization status of girls than boys as observed by Gupta et al (2016) was not because the community cared more for the girls, but because injections were not generally perceived to be safe for children, so they would let girls have them rather than boys [15]. Several client and community level barriers were thus observed in this study. These have to be addressed appropriately for an effective implementation of the NRHM health sector plans. Similarly, at the ground level, NRHM schemes were sometimes considered poorly visible indicating an information gap between service providers and users. Perhaps lessons can be learned from Taleb et al (2015) study in Bangladesh, where the maternal and newborn health improved by a focused and dedicated bridging of the information gap through community-based programs that influenced knowledge levels and practices of women [43].

Similar results as in our study are reported in some of qualitative studies done on the effective role of accredited social health activists in improving MCH in Uttar Pradesh [44], on barriers in the financial incentive scheme (like the need for having identity documents by pregnant women) in Madhya Pradesh [45], and on the poor functional status of village health nutrition and sanitation committees in Maharashtra [46] in India. However, gender-based barriers by the female health workers as observed by Mumtaz et al (2003) in Pakistan were not reported in this study [47].

The strength of this study is its integrated approach and holistic review of NRHM’s MCH plans. Further by comparing two extreme situations in two selected districts this study provides contextual information on what works and what does not work and identifies modifiable barriers at the health system, client and community level in the implementation of the MCH program that affect the accessibility, availability and affordability of MCH services and ultimately the MCH outcomes and inequalities. Like it was realized that social determinants of health like status of women in the society, education level of women and overall development of the district played a significant role in the proper implementation of the MCH plans of NRHM in district Mewat. The status of women was low in district Mewat, hence women could not take decisions regarding their own or their child’s health that made them dependent on their husbands or mother in-laws. There was also insufficient number of social health activists in Mewat, as many of the potential women could not meet the criteria of becoming health activists due to low education status of women. Hence, behavior change communication at the household level regarding utilization of MCH plans of NRHM was restricted to villages with social health activists. Since, Mewat was the least developed district in Haryana, not many service providers preferred to work in the health facilities located in this district. Other important issue identified was the cultural barrier in accepting the MCH schemes like immunization, which needed different community level strategy to increase the utilization. This information is crucial for a better planning and effective implementation of the program, not only in Haryana, but also in other states of India, and possible also in the second phase of NRHM (2013–17) as part of the National Health Mission [16]. These contextual factors or preconditions might be different in different districts. However, lessons learnt from one district may be transferable to other districts due to similar health system characteristics.

Based upon the findings of the study it is recommended to have district specific strategies to improve the implementation of MCH plans of NRHM. For district Mewat, involvement of local leaders and religious groups seems of utmost importance for increasing the acceptance of MCH plans especially immunization and family planning, and also involving them in increasing the education level of women at large in that society. Public health specialist should be appointed in all the community and primary health centers to assist the medical officers in implementing the MCH plans in Mewat, as lot of effort needs to be done at the community level to increase the utilization of MCH services, with continuous monitoring and supportive supervision of auxiliary nurse midwives and social health activists. Provision of stable manpower in district Mewat will further improve the implementation. Some of the common recommendations as also suggested by the stakeholders to overcome the barriers in both the districts include opening of bank account of pregnant women at zero balance and delinking it with identity proofs, providing career and financial incentives to doctors and paramedical staff working in difficult areas, maintenance of ambulances regularly, availability of more ambulances, alternative doctors (Ayurevedic/Homeopathic) in mobile medical units, increased financial incentives of social health activists.

Limitations of this study include those that are inherent to the qualitative study design, like results of this study may not be generalizable as health system gaps are to a large extent context specific. We acknowledge that this study was conducted in two districts of Haryana and among purposively selected respondents whose views may not represent the opinions of all community members, service providers or mothers. However, we believe that lessons learnt from this study may be applicable to other districts in Haryana and states in India because of the similar health care delivery system. Other limitation could be the bias in coding the qualitative data. However, two trained persons had analyzed the qualitative data independently, hence chances of such bias is minimal.

Overall, it can be stated that NRHM’s health sector plans have succeeded well in improving the MCH outcomes and in reducing geographical and socioeconomic inequalities in Haryana. However, to make a policy a success apparently is complex and, as was observed in the Mewat district, dependent on many interrelated factors, like political, economic and sociocultural factors. The policy implications of this study are that, along with the implementation of the specific MCH schemes, the structural determinants of health inequalities (education, occupation, income, socioeconomic status) and the basic socioeconomic development of the district need also to be addressed. Unless that is done, the extent and effectiveness of implementation of MCH plans runs the risk of remaining only a partial success. It is also recommended that MCH plans needs to be scaled up through an extensive availability of human resources, a reduction in the information gap between service providers and users, and an effective planning and implementation of the targeted interventions to deal with client and community-level barriers.

## Supporting Information

S1 File NRHM health sector plans. (PDF)

S2 File Focus group discussion and in-depth interview guides. (PDF)

S3 File Framework matrix showing examples of coded data. (PDF)

S1 Table Themes and codes as per applied thematic analysis. (PDF)
