# Supplementary material for: Impact of a Multi-Strategy Community Intervention to Reduce Maternal and Child Health Inequalities in India: A Qualitative Study in Haryana
Source: PLoS One. 2017 Jan 18;12(1):e0170175. doi: 10.1371/journal.pone.0170175 (PMC5242542; doi:10.1371/journal.pone.0170175)
Supplement: S1 File — (PDF) [file pone.0170175.s001.pdf]

**S1 File. NRHM health sector plans**

## 1. Health system strengthening

Infrastructure strengthening

Financial support is provided to States to strengthen the public health system including upgradation of existing or construction of new infrastructure. Under NRHM high focus states can spend upto 33% and other States upto 25% of their NRHM funds on infrastructure.

Drugs and logistics

States are being incentivized up to 5% of their total outlay NRHM to prepare policy and establish systems for free distribution of essential drugs including preparation of essential drug list, standard treatment protocols, robust procurement system etc for free distribution of essential medicines in Public Health facilities. Various programme components under NRHM such as Maternal health, Child health, Family planning, Adolescent health and National disease control programmes prescribe specific drugs which are either centrally procured or funds are provided to States/ UTs for decentralized procurement. Apart from programme specific drugs, funds are also provided for general drugs and supplies

Patients transport service

Patients transport service (Referral transport) patient transport ambulances operating under dial 108/102 ambulance services. 102 services essentially consist of basic patient transport aimed to cater the needs of pregnant women and children though other categories are also taking benefit and are not excluded. Free transfer from home to facility, inter facility transfer in case of referral and drop back for mother and children are the key focus of 102 service. 108 is predominantly an emergency response system, primarily designed to attend to patients of critical care, trauma and accident victims etc.

Human Resource

Financial support is provided to strengthen the health system including engagement of nurses, doctors and specialist on contractual basis based on the appraisal of requirements proposed by the states in their annual programme implementation plans.

Untied Funds, Annual Maintenance Grants and Rogi Kalyan Samiti (Patient welfare committee) funds

To promote functional, administrative and financial autonomy in the health facilities NRHM has provided provisions in the form of availability of untied funds, annual maintence grant and patient welfare committee funds at sub center, primary health center, community health center and district hospital mainly for repair of instruments and maintenance of buildings.

Mobile Medical Units

Mobile Medical Units are a mechanism to provide outreach services in rural and remote areas. This is not meant to transfer patients. Mobile Medical Units comprise of one/two or three vehicles varying state-wise. Where there is more than one vehicle then one vehicle is used for transport of medical and para-medical personnel, second is used for carrying equipment/ accessories and basic laboratory facilities, third vehicle carries diagnostic equipments such as X-Ray, ultrasound, ECG machine and generator. Each unit has- one doctor, one nurse, one radiologist (if available), one lab attendant, one pharmacist and a helper and driver. There is provision of free medicines in the unit.

**Maternal health care strategies**

Janani Suraksha Yojna (Financial incentives for institutional delivery)

Launched in April 2005. Cash incentives were given to pregnant women on institutional deliveries. In low performing states all pregnant women were beneficiary, in high performing states pregnant women belonging to below poverty line were the beneficiaries. Schedule caste women are beneficiaries in both types of states.

Janani Shishu Suraksha Karyakaram (free institutional delivery and sick infant care in the hospitals)

The initiative launched in June 2011 and entitles all pregnant women delivering in public health institutions to absolutely free and no expense delivery, including caesarean section. The entitlements include free drugs and consumables, free diet up to 3 days during normal delivery and up to 7 days for C-section, free diagnostics, and free blood wherever required. This initiative also provides for free transport from home to institution, between facilities in case of a referral and drop back home. Similar entitlements have been put in place for all sick newborns accessing public health institutions for treatment till 30 days after birth. This has now been expanded to cover sick infants. The scheme aims to eliminate out of pocket expenses incurred by the pregnant women and sick new borns while accessing services at Government health facilities.

**Child health care strategies**

For child health focus was given on providing newborn care units at the facility level by facility based new born care scheme, skilled management of under five illnesses by providing trainings of the doctors, staff nurses and strengthening of facilities under facility based management of childhood illnesses. Medical officers and ANMs provided community-based management of newborns and children illnesses through integrated management of newborn and childhood illnesses and home based post natal care. Malnutrition was targeted through approaches like infant young child feeding practices and establishing nutritional rehabilitation centers for severely malnourished children. Since mortality due to diarrhea and pneumonia is

very high among under 5 year olds hence targeted approach followed for these two diseases. Supplementation of micronutrients was also provided. Immunizations coverage improved in remote and rural areas through provision of alternate vaccinators and increasing the number of outreach sessions.

**Communitization**

Accredited social health activist

It included provision of a female accredited social health activist for each village who served as link between the community health care delivery system. She belonged to the same village and had basic educational qualification (upto eighth standard, but in district Mewat upto fifth standard). She was given performance-based incentives for providing antenatal, postnatal and child-care. She has to do the behavior change communication with the antenatal, postnatal women or with potential decision makers in the families for antenatal care, immunization, and institutional delivery, post natal care etc.

Village health nutrition and sanitation committees

Village heads were involved by forming village health and sanitation committees so as to prepare village health action plan as per the needs or problems in the villages. This committee has been sanctioned a sum of Rs 10,000 to perform the activities annually. It gets deposited in a joint account in the bank.

Village health and nutrition days

Maternal and child health services provided through organization of village health and nutrition days each month in the village where antenatal care and immunization of children were mainly done.
