# Supplementary material for: Impact of a Multi-Strategy Community Intervention to Reduce Maternal and Child Health Inequalities in India: A Qualitative Study in Haryana
Source: PLoS One. 2017 Jan 18;12(1):e0170175. doi: 10.1371/journal.pone.0170175 (PMC5242542; doi:10.1371/journal.pone.0170175)
Supplement: S2 File — (PDF) [file pone.0170175.s002.pdf]

S2 File. Indepth Interview/Focus Group Guide

Section 1: Informed consent taking

Section 2: Introduction about project (study information sheet provided)

Section 3: Extent of implementation of NRHM health sector plans for maternal and child health

- What is your opinion on NRHM plans/schemes for maternal and child health?
- What do you think is the status of various schemes implemented to improve maternal and child health under NRHM?

Section 4: Status of Maternal Health strategies under NRHM implementation

- What do you feel about implementation status of the existing maternal health strategies under NRHM?
- Do you feel that after NRHM plans maternal health has improved compared to the status before its implementation? If yes how?
- Does the death rate of mothers has decreased after implementation of NRHM? If yes how?

Section 5:Status of Child Health strategies under NRHM implementation

- What do you feel about implementation status of the existing child health strategies under NRHM?
- What is the immunization status among children? Are all children getting all vaccination in time? Do you think NRHM has helped in improving immunization status among children?
- Do you feel that neonatal care (Newborn) has improved after NRHM implementation? Could you describe how?

- Do you feel that after NRHM plans child health has improved compared to the status before its implementation?

Section 6: Accessibility, availability and affordability of maternal and child health services

- **Availability**:

  Do you feel that implementation of NRHM schemes, heath services of maternal and child health were able to reach rural areas, poor women and children and disadvantaged group?

- **Accessibility:** Do you feel that there is improvement in accessibility of maternal and child health services after implementation of NRHM schemes in rural areas/poor women and children/disadvantage groups?

- **Affordability:**

  Do you feel that According to you are people satisfied with existing NRHM schemes? NRHM schemes have done anything in making maternal and child health services affordable for people especially for people in rural areas and poor families?

Section 7: Status of maternal and child Health Inequalities after NRHM implementation

- Do you feel that health sector plans implemented under NRHM has influenced these inequalities in any way? If yes, how?

- Do you feel that this inequality with respect to maternal and child health among rich and poor is still there?

- Is there any improvement in these inequalities after introduction of NRHM?

- Do you feel there are differences in the health status of males and female children?

Section 8:

- Are people accepting NRHM and its schemes?
- According to you are people satisfied with existing NRHM schemes?
- Do you feel there are barriers in implementing NRHM schemes to reduce the geographic, socioeconomic and gender health inequalities in the district?
- If yes, according to you what are the possible solutions to overcome these?
- What do you feel is the key to success of the NRHM schemes?
