# Supplementary material for: Impact of a Multi-Strategy Community Intervention to Reduce Maternal and Child Health Inequalities in India: A Qualitative Study in Haryana
Source: PLoS One. 2017 Jan 18;12(1):e0170175. doi: 10.1371/journal.pone.0170175 (PMC5242542; doi:10.1371/journal.pone.0170175)
Supplement: S3 File — (PDF) [file pone.0170175.s003.pdf]

S3 File. Framework matrix showing examples of coded data.

**NRHM Health Sector plans**	**District**	**Program Managers**	**Senior Medical Officers**	**Medical Officers**	**Auxiliary Nurse Midwives**	**Accredited Social Health Activists (ASHAs)**	**Community Leaders**	**Mothers**
**A. Health system strengthening**								
Infrastructure	Ambala	Infrastructure has improved a lot, earlier it was negligible. Only after NRHM new born corners and stabilization units were established	There is lot of strengthening of infrastructure.	Infrastructure has improved considerably	Infrastructure has improved but still there is gap and that is because of manpower. 70% improvement has done with NRHM and pending 30% are because of man power.	There is lot of improvement in infrastructure. From past 3 years facilities have improved in primary health centers	There is lot of improvement in infrastructure .	Good government hospitals are there. Many facilities are being provided. Services are becoming better here.
	Mewat	there is lack of infrastructure in terms of equipments and manpower: "We have 10 PHCs and out of those only 7 have new born care corners and 3 PHCs dont have baby warmers.	infrastructure is good but without adequate manpower	NRHM has improved infrastrucutre a lot due to its funding.	infrastrucutre has improved than before. There are vehicles now for going for immunization.	Infrastructure has improved. There are facilities in government hospitals for mother and children.	Infrastructure has improved: because of ambulance improvement is there. In hospital proper care is given. "	At PHC mother said that facilities have increased from 2-3 years and infrastrucutre has improved, but in some village mothers said that nothing has improved
Drugs and Logistics	Ambala	There is less shortage of medicines	Free medicine supply is there in almost all the health facilities, this has increased the patient load in the hospitals, which is less likely to be managed by single doctor in	Free medicine supply is there	Getting free medicines is not an issue	Mothers feel happy after getting free medicines	villagers now go to public health facilities because they know they will get free medicines	we get free medicines from the facility

			the PHCs					
	Mewat	Free medicines are available	Medicine supply is continuous after NRHM	there is no shortage of medicines in Haryana	Availability of medicines have improved.	mothers usually ask her husband or mother inlaw regarding treatment of her children, who do not trust public health facilities, hence get it privately	most of the villagers prefer private treatment of their sick children. But now with improved facilities trend is changing. Some of them do go to public hospitals	we usually go to private health facility to get the treatment for my sick child. Medicines in government supply is not of good quality
Human Resources	Ambala	There is provision of hiring contractual staff to deal with the problem of shortage of manpower	We are getting staff because of it. But there is conflict between contractual and permanent staff. Contractual staff say that I am getting 10,000, why should I do more work. Load has increased, but there are less number of doctors to manage the load.	We don't have staff. Staff is very limited.	70% improvement has done with NRHM and pending 30% are because of shortage of man power.	Number of ASHAs are less in Mewat. There are not enough educated women here. Those recruited also face difficulty in understanding the job properly.	There are ASHA workers and doctors.	

	Mewat	Lack of manpower. Trained staff nurses are not there	Shortage of Manpower. If man power will be less, so indicators will also weak. ANMs are not here, like somewhere ANMs have 10 village and in some villages there are no subcenter (there are more expect actions but Manpower is not up to that level) there is population of 10 lakhs and sub centre are 84, out of that 84, 14 vacant. How will this gap be filled?”	There is serious lack of manpower and it affects working of NRHM. "Punhana is on the population of four and a half lakh. And there only 2 MO are there, and that is high risk area. How will it run on 2 MO? And you have to take care of causality there, and OPD and meetings are also there.”	There is shortage of manpower and work load is more. "we have one ANM on population of 12000 and someo n 15000".	Some doctors are careless and not taking much care of mother and children.	Buildings are made good but doctors are not there	
Referral transport	Ambala	Ambulances are rather the best system. They have given a very good back up. All the 99% institutional deliveries in which government share is maximum i.e. 50% is all because of Ambulances (102) and ASHA.	it is a good scheme but on other side they think that ambulance have broken down and they need replacement now. They said that no maintenance of ambulance is there and they are breaking down now.	Hype of institutional deliveries is because of ambulances but its implementation is not proper as it is not available to everyone as there is no maintainence and delay in reaching home.	Ambulance facility is a very good facility in reducing maternal and infant deaths. On asking what is the reason for infant death reduction one of the reasons given by ANMs was ambulance.	Ambulance is a very good facility. They also said that because of ambulances they can go with family and accompany mother.	According to community leader at PHC ambulances are working and they come whenever needed. Awareness should be more regarding ambulances.	Everyone was aware about ambulance service. It was availed by some mothers and it came immediately when it was called.

	Mewat	Institutional deliveries have increased due to referral transport. But there is shortage of vehicles. If vehicle will increase then there will be more increase in institutional deliveries.	Institutional deliveries have increased due to referral transport but mothers don't stay for 48 hours post delivery in the hospitals, which was basic objective of referral transport. Repair of off road ambulances should be priority because once they stop working no one repairs them.	Implementation is poor as call centres are most of the time busy and ambulances never reach on time. This service need some changes "Implementation is there, but specific requirement is not taken from here (In Mewat) (Vehicles are less). Connectivity is not in villages. Where the birth rates are increasing, availability should also increase".	There is shortage of ambulances. There were time when 4-5 deliveries were there but free ambulance was only one and ambulance used to get late and deliveries used to happen at home or on the way.	Availability if ambulances is less. Ambulances dont come on time. Nobody picks phone at ambulance call center. They dont come at night and come very less in day timing. Ambulances picks up but does not drop. They (health care providers) want mothers to stay for 3 days but mothers want to come back as it is hot there with lot of mosquitoes in the health facilities amd also because of other houshold responsibilities.	Due to referral trasnport children from nearby villages come and are able to avail health services. Earlier people were having home deliveries but now due to 102 awareness has come and vehicle reach when they come and drop as well.	some mothers availed ambulance and were satisfied but one of the mother said "I had delivery at my home as i called ambulance so many times but it did not come so had home delivery".
Untied funds	Ambala	Because of untied funds all the things and facilities are available up to sub centre level now like newborn corner for delivery. Also if a mother comes for delivery,	United is the best part. Its allocation is like you don't have hindrance.	With untied fund there is no shortage of medicines and everything is available easily	Untied fund helps in buying small things of subcenter but it is not sufficient.	According to ASHAs untied fund is used to attract mothers for meetings as tea is served with that money. One of the ASHAs		

		everything is available for her				said “Like they serve tea in meetings. No one comes without tea or something”.		
	Mewat	Untied fund are properly utilized. They are properly utilized and subcenters are supported with them.	Untied funds are not sufficient.	Untied funds are helping and very good implementation but needs clear guidelines: "There is no specific guidelines like how can we do expenditure with these funds? We ask with each other and then utilize these funds.”	untied funds are not sufficient. "We are not able to do work as we want to".			
Mobile Medical Units	Mewat		Implementation is poor as there are no doctors in it and because of its size it can’t reach villages”.	No role of MMU: White elephant. No doctors in them so what is the use and they are too big to go in far areas.			Do not know	No mother knew about MMU.

B. Communitization								
Accredited Social Health Activists scheme	Ambala	Because of ASHAs institutional deliveries have improved. It also has a very good impact because these women work at grass root levels. Any mother or children are identified by ASHA workers at grass root levels.	ASHA motivates them, because they are closer in contact than us because they belong to that village so they can explain better." "ANMs have become lazy after introduction of ASHAs. Now they think that everything should be done by ASHAs."	ASHAs have given lot of benefits : " ASHA is a very good step. All ANC are registered with the reason of ASHA. ASHA finds ANC out of her thousands population and registered them, give injection, that is why maternal health is good because if there is any case of Anemia ASHA works very fast."	ANMs concluded that in Ambala there is 100% reduction in home deliveries and it is because of ASHAs. ANMs complained that they face problem giving incentives to ASHA as their guidelines regarding payment are not clear to them. ASHAs are given trainings before them regarding changed guidelines creating confusion.	According to ASHAs they convince mothers to deliver in government facilities and in any problem they can call us. They also replied "Main thing is ASHA is from village. ASHA knows everybody and people feel comfortable with her. We are like friends or their sister to them". Another ASHA replied "ASHA knows about all village and pregnant mother. Sometimes women cannot talk things to doctors but we can talk to them. They tell us".	ASHA has door to door approach and that is why health is improved. There is lot of contribution in its improvement . ASHAs are doing lot of work. people do have trust on ASHA. They call ASHA in problem.	Mother from CHC level said that they are just for immuization and nothing else on other side mothers at PHC and Subcenter level were very happy with ASHA: ":"I ask everything from ASHA only."

	Mewat	Program is running good because of ASHA but there is shortage of ASHAs. Because of more illiteracy in women problem is comming in recruiting ASHAs and also they dont have fixed salary.(educational status of ASHA- recruitment policy, human resource management issues- no fixed salaries)	ASHA is working good and is filling the gap of shortage of ANMs but as they are illiterate so problem comes. "They are doing work but problem comes. She can't fill the voucher. They don't know hindi or English and coordinator has been appointed for ASHA but they are also not giving any help. They don't get timely payments due to which their efficiency is affected	ASHA has a very good role in increasing institutional delivery and immunization but it is not completely implemented because there are no ASHAs in some villages. There is gap of 260-280 ASHAs in district.(Increase institutional delivery, shortage of numbers (HR related issues)	Due to ASHA lot of difference has come. Now all ANCs are known and immunization status has also improved. ASHA also does HBPNC which has helped in improving child health. Earlier they were also helpful in increasing institutional deliveries but now their payment has stopped due to which home deliveries have again increased.	" There is more facility because of ASHAs in village". ASHA has role in immunization, HBPNC an giving awareness. But some peple are not accepting and tell hem to go.	Impact of ASHA is very good. They hep in immunization and accompanying mothers to health centres. ASHA is bringing mothers to hospital.	Mothers at CHC and PHC level knew about ASHA and infact they helped them a lot: "ASHA helped them a lot. Helps pregnant ladies and children in availing services. But mother at village level had differnt scenario. They did not know about ASHA. They said that none came at home for any checkups.
Village Health and Nutrition Day celeberations in the villages	Ambala	Program manager perception was that earlier it had good impact but now it has lessened as now work load is more with medical officers.	VHND has good impact because medical officers are very busy and usually on the day of VHND they are doing some other work: "we are pre-occupied with many things. Report has to be give on the day of program (VHND). On the day of nutrition	Medical officers concluded that it is unnecessary scheme. One of MOs replied: "It is a dump scheme. It is not so much highlighted. Like you talked about this matter but this is dump from starting days".	According to ANMs VHND is working and it is being carried out by ASHA workers and it is done on the same day as immunization day. VHND has a good impact and now mothers are made aware of all schemes.	VHND brings lot of difference to mothers' health. We tell them to eat green vegetables, eggs, juice and fruits	Community leader at CHC said that no such days are celebrated whereas community leader from village said that mothers are called and given information.	On immunization day information regarding nutrition is given. Some mothers attend and some dont know about it.

			we will be busy with some report".officers.					
	Mewat	VHND Is not implemented as focus is more on immunization as it is conducted on immunization day.	VHND is not running. Sometimes they do one session some day. Otherwise it is not done		VHNDs are conducted on immunization. Lectures are given to mothers on health topics. Now mothers themselves come and ask questions. It helped in increasing awareness	Village health nutrition days are conducted. Not much effect is seen because some understands and some dont. Iron is given t o ladies. Some eat them and some ladies throw the pills	VHND is not working much.	On Immunization day all ladies are informed to come. "It is organized by ASHA and everyone comes."
Village Health and Sanitation Committees	Ambala	communitization has not happened as people are not much aware at this level.	the funds of VHSC are not utilized properly from the time the account has come to anganwadi workers as they don't want to use the money.	VHSC is a dump scheme. They said that this scheme is not giving any benefit	VHSC is not working and its implementation is not good.they worked for few months then closed.	VHSC is doing good work. They are doing lot of work in village	VHSC meetings are held in villages. Mothers are told about immunizatio n and other thing in these meetings. Sanitation work is also done under this.	

	Mewat	VHSC are not properly implemented as they are not able to do expenditure.	Yes, they are made but they are not utilizing a single penny. In that problem is female Sarpanch don't want to go to banks. She needs fare of transportation. The money is lying like that and there is no use of that. The bank is far from villages. How will only one ANM, handle VHSC, VHND or another thing? There are so many things."	It has no role and function and that it should be stopped. They are not functional. They should stop it	VHSC is not working from the time charge is given to anganwadi workers. They have money from past 3 years but they are doing nothing. Sarpanch dont let them use it.	There are commitees and money comes to Anganwadi. Due to the mony now there is cleanliness in village.	Due to VHSC now there is cleanliness in village.	No mother knew about VHSC.
**C. Maternal Health Care Strategies**								
Financial incentive scheme for institutional delivery (Janani Suraksha Yojna-JSY)	Ambala	JSY was working very good till last year but from this year it is not working good because of delayed payments: "JSY was working very well till last year, and Ambala was among one of the best districts in terms of expenditure and beneficiaries. From this year we are facing problems	JSY is a good scheme and it helped in increasing institutional deliveries but it has certain drawbacks. It was said by one of the SMOs that "In JSY, major drawbacks are account number. There accounts have not opened. They	Good scheme: JSY is a good scheme and it is good now that money will directly go in their account as now they donot have to wait for ANMs to give them payment.	Implementation is not good. There was guideline in JSY that at the time of registration and at the time of delivery money will be given but now registration is done , delivery also and child is of 1 year still money is not given so what is the purpose of JSY"	JSY implementation is good and mothers get money on time. BPL comes to hospital with JSY.	JSY is a good scheme as due to financial help people are attracted to health centers. there is lot of improvement because of JSY" "all women are getting benefits under it. There are	According to one mother at village had delay in receiving money of JSY: "Son is 8 months old but still did not get money. First they were telling money would be given in Naggal bank. I filled a form for that. Then they told money will come in SBI bank now i have filled form

		because in Ambala and Sonipat incentives are now Adhar Card based".	don't have Id proof. Payments come after delivery. Problem is funds are not coming on time especially from state".				many benefits. Poor people were unable to have a good diet. Now they are having good diet as well."	for that".Frequent change of procedures/bank
	Mewat	Implementation of JSY is proper. Target is achieved of JSY.	No use of the scheme if the money is not given on time and this is a big hindrance. "Implementation is there but there is some delay in it. It is like justice delayed is denied; there is no utility of the money if they don't get it at registration. Payment are done by cheque, no account are there of ladies. It is a big problem that they are not opening account on Zero balance. Women don't want t to come outside for any reason. If they	Implementation is 70-80% and delay is due to cumbersome paper work: : "Maximum ladies have no account no. who will open account for Rs 500/or for1000. why not giving relaxation so that they can get money easily with minimum paper work"	Because of JSY institutional deliveries have increased. But there is delay in payments. Their children have completed one and a half year still money has not come. NRHM funds come but state funds come late.	Money is given to mothers but most of the mother are gettig home delivery and they also complain that in Rajasthan incentives are more	JSY has helped in increasing institutional deliveries but JSY is not successful due to shortage of doctors because even if they come to hospital there is noone to conduct deliveries. Delayed payments are because clerical department people do problems at all level.	JSY benefit was given: "Benefit was given of Rs.700 after one month". Very less mothers took benefit of JSY because majority mothers had home delivery.

			have to go Bank for 2-3 times, so they have to spend Rs 200/300/- for travelling only so problem comes.”					
Free institutional delivery and treatment of sick infant scheme in the hospitals (Janani sishu suraksha Yojna - JSSK)	Ambala	JSSK is very good and it is a very good scheme for maternal and child health as it helps in availing various services needed in emergency.	JSSK is a very successful scheme: "JSSK is a very successful scheme and it changed the scenario of maternal and child health."	JSSK is giving everything free to mother and children. It has helped a lot.	Due to free facilities mother and children are coming to government facilities. Mothers are not coming because we are giving money but they are coming because there are free facilities.	Home deliveries have decreased and one of the reasons for it is JSSK. This scheme is good and it is providing free facilities to mother and children	JSSK is giving help to mothers and children: “people are coming to hospitals as they don’t have to pay anything for delivery”. (Mobilized)	All mothers whether at CHC level, PHC level or Village level had free deliveries at government health facilities: "Yes I know about free deliveries. I know that diet is given to mothers and children. Almonds and dry fruits are given during stay in hospital"

	Mewat	It is completely implemented at GH and also at PHC, CHC. Food and free transport is given but need of vehicle is more to improve the implementation.	it has a good impact as everything is free during delivery. "Overall JSSK is working good. Medicines are free and diagnosis is there in that and there is blood. So it is a relief for ladies. So that there is no need to get things for delivery now. So that is a good impact. Institutional delivery is increasing because of this because now they know their delivery is free".	lack of manpower and infrastructure is resulting in poor implementation:" when staff checks mother's HB (then it is less) then they say, go to GH Mandikheda. If they have to do BT then they send them to gurgaon then people think that home delivery is better than that. If you will go to village they don't have trust in government. They think that they refer the delivery". " there is one gynaecologist and one paediatrician which affects the program."	In JSSK timely treatment is given in all problems. Mothers are made to stay till 48 hours thus making mother and child is safe. With JSSK life of children are saved.	free delivery is conducted and mothers get food to eat but most of the mothers prefer home delivery. Mothers are scared of hospitals: "Doctors of Mandikheda beat them so because of fear they prefer home delivery". Not much care is given to mother and child in hospital.	Under JSSK diet is given to mothers and tranport is also available. " If there is no doctor so JSSK will fail and it is already failing".	Majority Mothers did not know about JSSK. One of the mother mentioned" Food was given during delivery. Everything is free".
**D. Child Health Care Strategies**								
Immunization	Ambala	immunization program is doing well from the beginning. 90% children are immunized. Problem comes in urban and not in rural now.	Immunization status is good.	everyone is getting children immunized.	Immunization sessions are conducted. ASHAs call mothers and their children to be immunized.	Vaccination status is very good: "people call us for vaccination. Now people are giving immunization to their children. They call us when	immunization is going good and all children are covered.	Immunization is provided. ASHA visit their home regarding this. ASHA follow up whether our children are immunized.

						are you coming to give injection to our children. "		
	Mewat	Alternate vaccine delivery(AVD) has improved the status of immunization. The barrier is that population is more and ANMs are less. Still there are 2 villages where resistance is there due to culture but it has decreased then before.	Microplanning is done for immunization but basic problem is that population is more and ANMs are less. There are so many programs which ANM have to do.	AVD has improved immunization status to a large extent: "Earlier session starts at 11-12, Now session starts at 10'0 clock. ANM collects at cold chain point or on planning unit and gives vaccination from there and session starts."	Immunization has improved. ASHAs get incentives so they help in getting everyone immunized and now vehicle is provided on immunization day. But there are some children who are not fully immunized as once their children have fever after vaccination they stop bringing for next dose.	there are still mothers who deny Immunization. "They tell us to get out, you are getting salary that is why you are coming". They are scared because of pain and swelling after injection.there are more pregant ladies and children but they are not immunized. they are scared of injection.	Immunizatio n is done properly. All houses are covered and if somebody is left then they go again if somebody is left. Immunizatio n has imprived " we have announced in Masjid and took out Fatwa that immunizatio n is not bad and now everyone is doing".	Immunization status is good: "Immunization is given nearby. ANM calls everyone from home".
Integerated Management of neonatal and childhood illnesses	Ambala	IMNCI is not implemented much. Not much supervisory meetings are done under it	IMNCI is not working much as it should have been. ANMs are not able to do work.	it is not much implemented.	IMNCI is not doing much work.children are not coming much as parents think that not much facilities are there in subcenter.	HBNC visits are done and go to see newborns at home for 42 days.	ANMs are going house to house to check children	ASHAs come home to see children. They check weight and temperature and tells about immunization

	Mewat	IMNCI is working around 25% as very less staff has got training and that too was 3 years back. If ANMs are not trained then it cannot work properly. Focus is more on immunization.	IMNCI is not implemented yet. It is not much implemented. Home based is implemented but all ASHAs are not given trainings in that.	IMNCI is picking up from past 6 months to 1 yearbut there is serious lack of manpower. It is a very good program but problem is in implementation. "It is working but it is not good in subcenter as staff is very less trained at subcentre. OPD reporting is not ok. Out of 6 ANMs only 2 are trained". " ther is one SNCU and one paediatrician at GH who has 10 charges. we cant come facility based nor home based as whole time goes in meetings and trainingd".	HBPNC is carried out which helped in improving health of children less than 42 days. 60-70% newborns are properly taken care only 30% people deny as they have more children and they dont care. FBNC has also improved. "we have ZINC,ORS and medicines. earlier we had nothing."	Home based care is given to mother and newborn. HBPNC is done by mothers. For children not much facilities are at village level. Children are referred at GH which is far.	There are facilities and medicine for children but there is lack of doctors. There is lack in paediatrician.	At PHC and CHC Facility based care is given to children: "Child was having loose motion and he was brought to subcenter and ANM gave the treatment". HBNC is carried out in some villages and in some area HBNC is not carried out.
Maternal and infant Death Review	Ambala	Because of this gaps are being identified. We come to know about gaps in facility, man power or knowledge	100% reporting of MDR and IDR is there. Earlier it was not there. Proper reporting is done by ANMs and form has to be filled for IDR and MDR.		It helps in identifying the cause of the mortality.			

	Mewat	it is not implemented upto the level of district as there is problem of manpower.	Reporting is done and there is increased reporting now.	Earlier there was no reporting of death. So thanks to NRHM now maternal and infant deaths are reported.	reporting of deaths are done now.			
**F. Maternal health outcomes**	Ambala	NRHM has contributed in improving maternal health overall	Lot of improvement in maternal health: "Early registration are done .Mother is getting complete facility. ASHA is doing care, doing check-ups. Home deliveries are stopped .If they are Anemic they come to hospital, and get well. Earlier Dai were doing deliveries. She doesn't know anything so mother used to die."	maternal health has improved a lot and it is because of ASHAs.	Now there is difference. There is no death case in past 3-4 years:" Earlier there was anaemia but now it is decreasing because we give them medicine".	there is more improvement as compared to earlier in previous 5 years.	"in previous times we used to hear that women got delivery and died. Not in this time".	there is improvement: "yes, there is some difference, now ladies do care". "previously there was less knowledge among people. Women were forced to marry at earlier age. Now people living in rural area are also much aware".
	Mewat	Maternal deaths have decreased because home deliveries are less. Now reporting of maternal deaths is more due to improved maternal death review systen but still it is more then state.	It has changed. It has improved. Improvement is there in the maternal mortality. If there is 40% registration then atleast 40% is saved. At least, there is growth in Economy	Maternal health has improved but still there are barriers to assess maternal health services (socioeconomic status, religious barriers, nutritional status, lack of manpower	Maternal mortality has decreased than before. "Institutional deliveries and immunization have become good. We heck for anaemia and if we will finish the cause then mother will be saved. But still unauthorized and not qualified people	Maternal health has improved as there are less deaths now and reason is immunization. Tetanus injections and iron capsules help in improving	Maternal health has improved now both child and mother are safe. Mortality has decreased due to decreased home	

			with NRHM. People hasve saved more money due to NRHM		are edoing deliveries which lead to maternal deaths.	health of mothers.	deliveries.	
**G. Child health outcomes**	Ambala	NRHM because of its facilities like SNCU and newborn corners it has decreased infant deaths.	there is lot of improvement then before. Now there is more awareness among people.	Due to HBNC there is improvement in health of newborns but it is for 72 days and nothing is being done for children after that. Not much work is done for under 5.	all ANMs positively answered that there is significant improvement in child health: Earlier there was 10-12 infant death. Now there are two. But ANMs said that not much is done for children after 42 days and IMNCI is not working properly.	child health has improved a lot. Mow mothers are also more aware regarding immunization and vaccination. HBPNC and checking children and mothers at home helps in improving infant health.	There is big difference in child health. ASHA goes door to door and make mothers aware. Mothers have started taking their chidren for complete immunizatio n	children do not fall sick much now. The only suffer from temperature. This is because of various facilities of government".
	Mewat	infant death is more than state but it has improved then earlier because of increased institutional deliveries , SNCU, Newborn corners and immunization.	Child health has also improved in mewat. There is lot of improvement due to SNCU. Referral transport and ASHAs also helped a lot.	There is decreased mortality and morbidity due to NRHM but problem is lack of manpower and no family planning. "“Danger signs are identified and referred. But Doctors are not there at PHC.	Infant deaths have decreased. It is mainly because of HBPNC. With the help of JSSK children who died with the reason of diarrhoea, ARI or with malnutrition are saved.	Chils health has improved as there are less mortality and it is because of immunization. HBPNC also is done and all visits are carried out.	Earlier children were dying but not it has changed. Immunizatio n is given to all the children. But one community leader said there is no	

				Sometimes they are on tour or in meetings. Than patient come to district hospital but he is not finding any doctor in hospital as there is only one pediatrician".			improvement in chid heath:" Mothers heres have no gap between chidren thus leading to poor health of children".	
**H. Access to MCH health services**	Ambala	Previously after 3-4 pm PHC were closed and due to this there was a decrease in accessibility. But now due to 24X7 PHCs accessibility has improved.	Accesibility improved a lot. More improvement is there with ambulance. All are getting facilities. Now, no problem is there. Even people know, they will immediately call on 102 thus improving access. People from brick kiln are also coming to take services.	OPD has increased due to ASHA. Because ASHA goes to village and send. She is the advertisement of our PHC. Accessibility is there and it reflects. Earlier people did not know about PHC , CHC or there is hospital but now she is our representative and does the advertisement.	Accessibility has improved due to improved infrastructure: "Accesibility has improved.Sub-centers are improved through NRHM. 70% infrastructure improved".	Accesibility is there: "health centre is open till 24 hours".	Accesibility has improved. Now people are coming to PHCs and CHCs.	accessibility has improved a lot especially for mothers as they doctors are available in the government institutions and ASHAs help them to seek care. But there is delay in treatment due to huge rush".

	Mewat	Accessibility is less due to lack of education and awareness. There are certain cultural barriers like some villages resist immunization.	Even if staff is available then accessibility is not there due to trainings and unnecessary reports." Cultural barrier also play a role in decreased accessibility. "Too much population, Mewat have too much programs and too much reporting. Even if ANMs are available, they are too much occupied in other activitities (trainings and reports) that they are not able to provide the services"	Location of health centers are not accessible. They are not at strategic location. There are cultural barriers as well. Even if doctors are available there are not accessible because either they are in training or in meeting. "medical officers dont have time for OPDs. we have so many death- birth. we dont do the work we have to do but we are doing clerical work more."	Accesibility is less due to illiteracy and cultural background. Old people of mewat refuse to send women outside. Some women dont want to avail facilities.	Though services are available but people are accessing due to cultural barriers. : "Muslim ladies deny immunization. They say it is not considered good in our religion". Due to carelessness of doctors people are loosing trust and thus not accessing the services.	Accesibility is less as there are still cultural barriers and families are not coming out to avail services.	Accesibility is not there: "To see a doctor have to travel 10 kms". Mothers at village level prefer home delivery as they are scared of hospitals and doctors. They think operations are done in hospitals and they are scared of that.
**I. Availability to MCH services**	Ambala	Availability has improved a lot. Previously also availability was there but we have noticed a lot of advancement in rural areas. Previously rural population was not coming for institutional deliveries but it has improved a lot now.	Availability has improved but there is lack of manpower and specialists: "Load has increased, but there are no doctors to manage. Paramedical are not there.LT should be more."	There should be more availability. There is only one sub-center on the population of 10000. And there is one PHC that is on 45,000 populations. So, number of MOs. should increase	there is still lack of availability due to manpower: "lack of man power results in lack in accessibility. People are coming but can't take facilities because of no manpower. If man power is complete so I don't think so then there will be problem in work".	Many health facilities which were not available earlier are available now.Doctors and ANMs are available easily. They refer the case in problem.	At all levels availability is there. All services are available now.	Availability has increased.No mother complained of non availabilty of services. One mother whose child was elder she said "during my pregnancy and delivery no one came but now ASHAs are coming"

	Mewat	Availability has improved due to ANMs as they are going to public and they are making them aware. But shortage of manpower is barrier to mothers for availaing services.	Availability is not there in terms of manpower.	Availability is not there due to lack of manpower:" there is one gynaecologist in whole distict". "We have vehicles at GH but drivers are not there."	Availability of services and medicines have improved but there is shortage of manpower.	There are facilities available for mother and children but at village level there is gap. Mothers have to travel far for delivery.	there is shortage of manpower as staff is not complete. "there are 30-40 deliveries and doctor is one. This is main problem".	Availability has improved with respect to ANMs and medicines but availability of ASHAs are not available in some villages.
**J. Affordability of MCH services**	Ambala	services are totally free now deliveries are also free , child kits are free , surgical package under one roof is available now.	Affordability has improved: "Yes, it is free. All is free of cost. Charges are low".	Affordability definitely increased. Expenditure is almost nill. Medicines and delivery all is free.	Affordability has improved: "everything is free. All medicines are available at PHC".	affordability has definitely increased.. Now everything is free so there is no problem in availing services.	Affordability has improved "Services are for poor people". "NRHM has a hand as people are getting free services. If someone had to go caesarean in private hospital then he has to pay 20-30,000 but it is very less in government hospitals. Free delivery is a big deal".	Affordability has increased: "people are getting more facilities in government hospitals. They are going less in private hospitals as tests are free now in government hospitals". According to one urban mother "now it is good in civil hospital than previous. Earlier we had to pay for the tests and now all tests are free ."

	Mewat	Affordability has improved. Services are free due to JSSK. All is free of cost.	Services are free of cost. All medicines are almost available in Mewat . They don't write medicine from outside.	Affordability is absolutely there. Govt is giving all medicines. All things are available.	Affordability is there. Every thing is free in government.	though treatment is free but they go to some private and borrow money: "they take from here and there. Due to negligence thet dont go to avail free services".	Services are affordable as now services are free and good. People here dont want to spend on their children and free services have helped them a lot.	"Treatment is free. Money is only spent on tranportation".
**K. MCH inequality in urban and rural areas (geographical inequality)**	Ambala	The gap is bridged: "In fact registrations are more in rural then in urban area. Now because in rural areas ASHAs and ANMs are available. So they ensure 100% registration. Focus is more on rural than urban and as a result staff is also less in urban areas".	It is bridged. Gap is decreased Now villagers are concerned same as urban people. It might be possible that urban people don't come but rural people definitely come. Rural people are coming more.		It is because of NRHM. Gap has decreased due to NRHM. There are less facilities in city hospital as compared to PHC	Gap is bridged: "Delivery hut are there in villages now. Medicines and injections are available in hospital and dispensary. There is an ambulance and available for all problems, treatment is giving to all. Everybody is getting all facilities in villages".	Gap is bridged: "“There is difference now as no one is going to cities. All facilties are in villages now”.	Different percptions were there. CHC mother said"“In cities there are lot of facilities, what is there in the villages?” But other mothers felt that there are increased facilities in villages: "services are better now. Previously we had to go to city for medicines, now everything is available here”.

	Mewat	In mewat no such demarcation is there between urban and rural. But in comparision with other disticts difference has come but it is slow due to shortage of manpower.	The area is mostly rural in mewat so no specific demarcation between rural and urban area.	There is no clear cut difference in rural and urban area in mewat but in comparision with other district there are inequalities though NRHM is trying to bridge: "Definitely. Like there are less Home deliveries, in immunization there is difference with immunization; institutional delivery is doing with good method."	The gap has reduced and now all facilties have reached in villages. It is because of ambulance also. Earlier facilities were in cities but now they have reached villages and it is due to NRHM.	there is reduced inequality. Facilities have slowly come to villages but for delivery mothers have to go to GH from villages.	Reduced as now health centres are in villages. "Buidling system has started like cities. Women are coming out and this is because of NRHM. Delivery huts of NRHM has given lot of benefit.	There were different perceptions: "Awareness has improved in vilages." "There are differences in villages which do not have facilities like cities". " "Earlier we had to go far for medicine but now we are getting everything in villages".
**L. MCH inequality between rich and poor (socioecono mic inequality)**	Ambala	Trying to bridge the gap: "Because of NRHM schemes, mothers come at CHC for deliveries by using 102 ambulance services. Earlier they had unsafe deliveries there only. Proper immunization sessions especially for them are also done now and all the children are covered under it".	gap is still there but it is because of personal differences as poor person is still poor and there is difference in diet of rich and poor but still facilities for poor has improved: "But there is a hope for poor. Diet is there for poor. Free ambulance, now they feel that they should go to the hospital. Now	This is bridged as now due to ANMs and ASHAs as they are available and they dont have to waste time to come. Earlier they were not coming for minor problems but now they are coming soon. So improvement in health is overall.	There is change in this gap. Rich were taking care, poor were not taking care. Now there is difference in early registration of pregnancy and that's the reason of improvement. If we give pills of HB for two times, then anaemic mothers also recover. This helps both children and mother. So, this gap reduced in between Rich & Poor and that too a lot	free treatment is given. There are tablet for Anemic and we request them to eat green leafy vegetables. Good care has been given to pregnant lady so her baby becomes also healthy gap of rich and poor has finished. Poor child is completely healthy and weight is also okay.	Mixed perceptions were there from community leader: "mothers receive diet for a week or 10 days but after that they have dry rotis". According to community leader PHC "Now children from poor families are also happy."	All the mothers had different perceptions: ""I don't think that gap is bridged". : PHC mother said" there is lot of improvement. Free medicines are given and poor people do visit government hospitals. Now delivery is also conducted here. All facilities are free now". According to one mother at village level this situation or gap has not

			infrastructure and cleanliness is better than before. Difference is not completely gone but there is improvement".				According to community leader at village level now there is no such difference.	changed. She thinks government should do something for poor.
	Mewat	Socioeconomic inequality is bridging but it is still there as people still do not have faith in government facilities and prefer private where they have to spend money and manpower is also less	According to SMOs mostly people are poor but here population is more and backwardness and illiteracy leads to this inequality.	Inequality is decreasing as IMR and MMR has decreased due to NRHM.	Lot of difference has come with NRHM. Earlier there were deaths but now its has stopped. JSSK has helped in bringing about the difference.	the ineqality is decreaased: " Earlier children were weak and mothers too but now there is difference. Medicines and Iron is given to them".	Inequality has reduced to quite extent in mewat: ow ASHA comes and tell mothers and they eat good food. Now children are of three and a half Kg. "People cant spend money on children in mewat thus free services have helped a lot."	Socioeconomic inequality has decreased: "People now know of free service and people who donot have money are going". "people were not bringing their children due to financial problem but now they are bringing.
**M. Child Health inequlity between male and female children**	Ambala	NRHM as such is not having any scheme on gender sensitization. As a whole, there is lot of improvement in community. A lot	NRHM specifically is not doing anything on this. There is little change in it because of	the inequality has decreased but NRHM has no direct involvement, it is because of awareness.Not	ANMs there is still difference but it has decreased quiet a lot:"If two girls, then they give money, but money is not the solution of any	this gap has been bridged. There is no difference now. Families are small now and have one	Gap is bridged because of ladli scheme and now there gender is not known	All mothers said no such inequalities exist now.

**(gender inequality)**		of people are not making any difference between girl and boy.	education and small families and not because of NRHM	Much is done on gender sensitization. Counselling of married couple should be done.	problem. But now people have awareness".	boy and one girl. They also said that ladli scheme and other schemes for girls help in decreasing this gap	before delivery.	
	Mewat	There is no such inequality in mewat.	there is no such inequalities as people here love girls more and they have so many children so they don't do any such inequality.	No difference in Mewat and it is by default and NRHM is doing nothing in this. Here people dont discrimnate between male and female	yes such inequalities are there. Some families do difference between boy and girl. Sometimes they give injection to girls not to boys that if girl dies its ok but boy should be safe.	there is no such inequality:"they are giving more care to girl."	there is gender inequality and problem is population:" if there are 3 daughter and 2 sons obviously they will care about sons." but other community leaders said inequality has decreased.	it has decreased and now there is no such inequality.
**N. Barriers in the implementation of NRHM**	Ambala	lengthy reporting system was a barrier as due to lengthy formats of HMIS they are not able to devote much time to program. Other barrier faced by program manager was that recruitments take lot of their time. They want NRHM to do recruitments for them.	lack of manpower and beds in hospitals is a barrier in implementation of maternal and child health services. They also complained that there is lot of reporting and some reports are asked again and again, already they are overburdened	Shortage of manpower is a barrier. Ambulances have broken down and they need repair.	1) Guidelines regarding payments of ASHAs are not clear. 2) There is gap in coordination between ASHAs and ANMs. 3) Overburden of work is there and it affects their working 4) Payment is not there for sweepers and electricity bill.	All ASHAs think that they are not paid properly in spite of so much work	there is lack of awareness and advertisements.	

			and this increases it more.					
	Mewat	1) There is shortage of manpower. 2) Vehicles of 102 should be more 3) Education level is less and it becomes a barrier. 4) 90% staff is of NRHM and there is no regular staff here unlike other districts	1) Main barrier is shortage of manpower.<br>2) Incentives are not the solution.<br>3) Mothers are not ready to stay for 48 hours.<br>4) There is too much population in mewat and on top of that there are too many programs and too many reports.<br>5) Family planning is acceptance is low due to cultural barrier.	1) There is acute shortage of manpower due to which medical officer are overburdened.<br>2) Subcenters are not at strategic locations. PHCs and subcenters are not easily accessible to people.<br>3) No acceptance for family planning is a big barrier for improving maternal and child health.<br>4) Ambulances are less in Mewat.	1) Due to no incentives for ASHA of delivery home deliveries have increased. 2) There is lot of reporting and some reports are asked again and again. 3) Manpower is less. 4)Funds for JSY are delayed 5) Ambulances are less.	1) Ambulances are not available when they are called.<br>2) people still dont know why ASHA s are there so they hesitate to accept them.<br>3) Cultural barriers are there as they think immunization is not in their religion.	1) Specialists are not there like paediatrician.<br>2) illiteracy is a huge barrier	

**O. Future suggestions**	Ambala	1) Manpower should be increased.<br>2) Repair and replacement of Ambulances (102) should be done.<br>3) Focus should be in urban area now as not much maternal and child health facilities are provided in urban area.	1) Manpower should be increased especially staff nurses and doctors.<br>2) Requirement of driver and vehicle is there for them for official purpose.<br>3) Repair and replacement of ambulances is needed. In ambulances with driver one helper should be there.<br>4) ANMs should be trained more to conduct deliveries.<br>5) VHND should be on some other day then immunization day.	1)Manpower should be increased. 2) Quality staff must be there. 3) Salaries should increase and continuous assessment of staff must be done. 4) Incentives should be given to all and not only to SCs.	According to ANMs they are overburdened with unnecessary work and their efficiency suffers. They also suggested that there are lot of reporting and there are Performa which are unnecessarily lengthy and they are not able to do their practical work. There should be fixed guidelines and reports.	1) Big hospitals should be in village so that people don't have to go outside.<br>2) ANMs should be more. There is only one ANM for 7 ASHAs. People feel insecure to come to sub center as there is only one ANM so they prefer PHC.	1) There should be proper advertisemen t at doorstep of people.<br>2) Increase facilities at CHC level like specialized doctors.	Whenever a lady gets pregnant they should come and tell all ladies what government is doing for them and what schemes are there.

	Mewat	1) Referral tranport facilities should be improved. 2) Manpower which is stable and not migratory should be recruited 3) more awareness should be given to public.	1) Either manpower should be increased or incentives should be more. Incentive should be activity based.<br>2) Create managerial post and doctors should not do work of management.<br>3) JSY money if given immediately after delivery will motivate mothers more.<br>4) AC vehicle should be there to pick and drop staff from gurgaon. This will improve status of Mewat and problem of manpower.<br>5) Vehicle should stand in PHC and then they will take everyone for field and they will go to villages and come back so services will be available in villages to	1) Incentives should be given to everyone right from ASHAs to all staff.<br>2) Subcenter environment should be secure for females. Class IV and chowkidar should be there. There is need to strengthen manpower.<br>3) Vehicles should be provided to nodal officers and one vehicle should be at PHC to increase accessibility.<br>4) Quality manpower should be provided.<br>5) Account assistants should be provided as medical officers feel they are not competent enough to manage account books.<br>6) Ambulances should be more according to the needs of the area.<br>7) Some relaxation should be given in JSY so that money can be given with	1) RMPs should be given strict instructions that they should not accompany Dais to conduct home deliveries. 2) Mulla-maulawi should be met and help in reducing cultural barrier. 3) Salary of ANMs should increase. Incentives should be given to ANMs as well.	1) Doctors should come in villages and address mothers and their families regarding immunization and tell everyone about ASHA so that people dont deny the services. Fear of immunization should not be there.	1) Lady doctor should incraease 24 hours. 2) ASHA payments should not be stopped. 3) ANMs and ASHAs should increase.	People in villages should be made more aware and they get support from government.

			people and then no need of sitting in subcenters.<br>6) PHCs should be given more facilities.<br>7) Nodal officers should be recruited at all subcenters and they should train ANMs as they don't know much work. Nodal officers should accompany ANMs and train them for one month.	minimum paperwork.				
